# Nitrite of Amyl

**Published:** 1878-04

**Authors:** 


NITRITE OF AMYL.—We take the following from an account of the work of Dr. Jvan Ermesagem on Nitrite of Amyl, in the above journal. "The author divides into four classes the diseases in which the nitrite of amyle may be used: 1st. Syncope, coma characterized by weakness of cardiac innervatiou, anæmia, and the venous congestion of the cerebro-spinal centres. 2d. Diseases characterized by vascular spasm. 3d. Spasmodic affections of voluntary and involuntary muscles, diseases characterized by extreme elevation of temperature. The nitrite of amyl is chiefly administered by inhalation. Three drops on a handkerchief will avert threatening syncope from chloroform. In sea-sickness it will succeed heroically, according to the observation of Dr. Clapham (a hundred per cent). In hemicrania, two drops will suffice to cure; but it is especially in angina pectoris and in asthma that the best results are obtained. But its employment is contraindicated in old people, or in those presenting any vascular or cardiac lesion. It is also contra-indicated in puerperal plethora. Its use at all times demands much circumspection."—*Rivista Clinica di Bologna.—Maryland Med. Jour.*

